# Immunotherapy Safety in Thymic Epithelial Tumors: Disproportionality Analysis of the Food and Drug Administration Adverse Event Reporting System

**DOI:** 10.2196/76908

**Published:** 2026-02-25

**Authors:** Ruilian Chen, Hanrui Chen, Lingling Sun, Yang Cao, Lizhu Lin

**Affiliations:** 1 Oncology Center, The First Affiliated Hospital of Guangzhou University of Chinese Medicine, 16 Jichang Road, Baiyun District, Guangzhou, 510405, China, 86 020-36596360; 2 The First Clinical Medical School of Guangzhou University of Chinese Medicine, Guangzhou, China; 3 Guangdong Clinical Research Academy of Chinese Medicine, Guangzhou, China; 4 Lingnan Medical Research Center, Guangzhou University of Chinese Medicine, Guangzhou, China

**Keywords:** immunotherapy, immune-related adverse events, thymic epithelial tumors, myositis, myocarditis

## Abstract

**Background:**

Immune checkpoint inhibitors (ICIs) have revolutionized cancer treatment, but their safety profile in patients with thymic epithelial tumors (TETs) remains poorly characterized due to the rarity of these malignancies.

**Objective:**

This study aims to comprehensively analyze immune-related adverse events (irAEs) profiles in patients with TETs using real-world pharmacovigilance data.

**Methods:**

We conducted a retrospective analysis of the US Food and Drug Administration Adverse Event Reporting System database from the first quarter of 2016 through the fourth quarter of 2024. Cases of TETs with ICI-related adverse events were identified and deduplicated following Food and Drug Administration recommendations. Disproportionality analysis was performed by calculating odds ratios, using the entire US Food and Drug Administration Adverse Event Reporting System database as the reference cohort. Signals were defined as significant with at least 3 cases and a lower 95% CI exceeded 1. Time-to-onset analysis and Weibull Shape Parameter testing were used to characterize the temporal pattern of irAEs. Clinical characteristics between fatal and nonfatal cases and cardiotoxicity specifics were analyzed descriptively.

**Results:**

Among 152 eligible TET cases with irAEs, males slightly predominated (80/152, 52.6%), with a median age of 58.5 years. Reports originated predominantly from the United States (51/152, 33.6%) and Japan (16/152, 10.5%). PD-1 inhibitors were implicated in 66.4% (101/152) of cases. Disproportionality analysis identified 14 significant irAE signals across 11 System Organ Classes. Myositis (reporting odds ratio [ROR] 113.15, 95% CI 26.65‐480.34), myocarditis (ROR 10.96, 95% CI 5.70‐21.07), myasthenia gravis (ROR 3.86, 95% CI 1.86‐7.85), and febrile neutropenia (ROR 18.33, 95% CI 4.57‐73.55) demonstrated the strongest associations. The median time to irAEs onset was 21.0 days, with 73.2% (41/56) occurring within 2 months of treatment initiation. Fatal outcomes were reported in 23.7% (36/152) of cases and were significantly associated with gender distribution (*P*=.04) and different treatment strategies (*P*=.01). The utilization of PD-1 inhibitors was higher in the fatal group (29/36, 80.06%) than in the nonfatal group (72/116, 62.1%). Myocarditis was the most frequent cardiotoxicity, accounting for 51.3% (20/39) of cardiac events.

**Conclusions:**

This large-scale pharmacovigilance study delineates a distinct and severe irAEs profile for ICIs in patients with TETs, characterized by robust disproportionality signals for myositis, cardiotoxicity, and myasthenia gravis. As a hypothesis-generating analysis, these findings underscore the need for vigilant monitoring and early detection strategies to mitigate irAE risks, particularly in high-risk subgroups such as patients with thymoma. The results provide clinically relevant evidence to guide risk-benefit evaluation and inform tailored surveillance protocols during ICI therapy in this population.

## Introduction

Thymic epithelial tumors (TETs), originated from thymic epithelial cells, are categorized as thymomas, thymic carcinomas, and thymic neuroendocrine tumors according to the World Health Organization [1]. TETs encompass a group of rare malignancies but constitute a significant proportion of anterior mediastinal tumors [2]. For early-stage TETs, curative surgical resection is the standard treatment strategy, yielding promising outcomes for a subset of patients. For those with advanced TETs, platinum-based chemotherapy has been considered as the first-line treatment [2,3]. However, there is no standard therapy for patients who experience disease progression after the first-line treatment of platinum-based regimens. Antiangiogenic drugs, mammalian target of rapamycin inhibitors, or other chemotherapy may be the potential option, although their effectiveness in this context remains uncertain [4,5].

Immunotherapy has gained great promise in the treatment landscape of various cancers, which sparked a significant shift in the paradigm of treating oncological malignancies [6,7]. The efficacy of immune checkpoint inhibitors (ICIs) was considered with a close relationship to the expression of programmed death-ligand 1 (PD-L1) [8,9]. Notably, TETs have been reported to exhibit high PD-L1 expression, with positivity rates ranging from 23% to 92% [10]. Encouraging clinical benefits have been observed with ICIs such as pembrolizumab, nivolumab, avelumab, and atezolizumab in patients with advanced thymic carcinoma or thymoma [11-14]. A phase II clinical trial demonstrated that the objective response rate (ORR) of pembrolizumab in 41 patients with thymic carcinoma was 23%, with a median progression-free survival (PFS) of 4.2 months and a median duration of response of approximately 3 years [11,12]. In the PRIMER study, the median PFS for nivolumab was 3.8 months, and the 12-month overall survival rate was 60.0% in a cohort of 15 patients with unresectable or recurrent thymic carcinoma [13]. In a study of 7 patients with thymoma treated with avelumab, an ORR of 57% was reported [14]. Despite these advancements, the unique immunologic environment of the thymus, which plays an essential role in T-cell maturation, raises concerns regarding immune-related adverse events (irAEs) [15].

Although the spectrum of irAEs associated with ICIs has been reported in various malignancies, the safety profile in TETs remains poorly defined [16]. This represents a critical knowledge gap, as the thymus is a primary lymphoid organ central to immune tolerance. The administration of ICIs in this immunologically unique context may disrupt central tolerance mechanisms, potentially leading to a distinct and more severe irAEs profile. Evidence from small-scale studies had shown a high frequency of severe toxicities in patients with TETs receiving ICI therapy [17,18]. Therefore, a TET-specific pharmacovigilance analysis is essential to systematically delineate these unique safety signals and inform clinical management. The US Food and Drug Administration Adverse Event Reporting System (FAERS) database provides real-world data on a global scale, while disproportionality analysis offers a validated method for detecting drug-event associations, especially for rare cancers such as TETs, where clinical trial data are limited. This approach effectively addresses evidence gaps by identifying clinically relevant toxicity signals and generating actionable hypotheses for risk management.

To gain a deeper understanding of the adverse events (AEs) associated with the therapy of ICIs in patients with TETs in clinical practice, we conducted a systematic analysis using the FAERS database to statistically evaluate irAEs in this patient population. This research endeavor aims to provide valuable insights into optimizing immunotherapy strategies for TETs while mitigating potential adverse effects.

## Methods

### Study Design and Data Source

This study was designed as a disproportionality analysis of individual case safety reports extracted from the FAERS database [19], covering the period from the first quarter of 2016 through the fourth quarter of 2024. The study was conducted and reported in accordance with the REporting of A Disproportionality analysis for drUg Safety signal detection using individual case safety reports (READUS) guidelines for studies using spontaneous reporting systems [20,21]. The primary analysis used disproportionality methods to detect significant associations between ICIs and irAEs in patients with TETs. Sensitivity analyses included stratification by ICI subclass and TETs histologic subtype. Additional methodological components comprised case-by-case clinical review of cardiotoxicity reports and contextualization of findings within the existing scientific literature. All AEs are coded using the preferred term (PT) according to the international MedDRA (Medical Dictionary for Regulatory Activities) (version 26.1). Detailed descriptions of the FAERS database and MedDRA structure are provided in Multimedia Appendix 1.

### Identification of ICIs Reports With Adverse Events

Our comprehensive analysis was conducted in 4 steps to identify AEs linked to ICIs in the patients diagnosed with TETs. Initially, we performed a deduplication process according to the Food and Drug Administration’s (FDA’s) recommendations in the FAERS database. For reports sharing the same Case Identifier (CASEID), the latest FDA Date (FDA_DT) is selected, which contained the most comprehensive information. If the CASEID and FDA_DT are the same, we retained the higher Primary Identifier (PRIMARYID). In addition, an extra deduplication step was performed by excluding cases that had identical gender, age, reporting country, event date, AEs, and prescribed drugs. Second, we extracted data for patients diagnosed with TETs in the INDI files. Our focus was on ICIs reported in the DRUG files, including programmed death-1 (PD-1) inhibitors (nivolumab, pembrolizumab, sintilimab, penpulimab, tislelizumab, toripalimab, camrelizumab, and cemiplimab), PD-L1 inhibitors (atezolizumab, avelumab, and durvalumab), and cytotoxic T-lymphocyte–associated antigen 4 inhibitors (ipilimumab and tremelimumab). Reported drug names were mapped to their corresponding active pharmaceutical ingredient names using the FDA’s National Drug Code directory and manual verification. For ICIs, both generic names and common brand names were unified under their respective active pharmaceutical ingredient terms. Age, gender, and country fields were normalized to FAERS conventions. Only reports in which ICIs were classified as the primary suspect were included, as the AEs in these cases were deemed most likely to be associated with the ICIs compared with other medications. Third, we extracted all reports containing AEs from the REAC files. Finally, we identified ICIs reports diagnosed with TETs with AEs by intersecting the PRIMARYID.

### Signal Mining

A disproportionality analysis is frequently conducted in pharmacovigilance research to identify safety signals and develop hypotheses concerning potential causal links between drugs and AEs. The reporting odds ratio (ROR) is used to compare the odds of reporting a specific event related to a particular drug against all other events while considering the reporting odds for other drugs within the FAERS database. The ROR was selected as the primary metric for this analysis due to its computational simplicity, transparency, and widespread adoption in the pharmacovigilance literature for initial signal detection. In this study, the ROR was used to identify signals of AEs in reports associated with ICIs in the patients experiencing TETs. By using AE reports from the complete FAERS database as a reference, we conducted a disproportionality analysis to evaluate the potential association between AEs and ICIs through the calculation of the ROR. The ROR and the 95% CI were calculated using the following formula:


$$ROR=\frac{a/c}{b/d}$$

$$95\%CI=e^{\ln\left(ROR\right)\pm 1.96\sqrt{\frac{1}{a}+\frac{1}{b}+\frac{1}{c}+\frac{1}{d}}}$$

a: Number of reports with both the target ICIs and the target events; b: number of reports with the target ICIs but without the target events; c: number of reports with the target events but without the target ICIs; and d: number of reports with neither the target ICIs nor the target events.

Following established pharmacovigilance practice aimed at balancing sensitivity and specificity, we defined a significant signal as one with at least 3 case reports and a lower limit of the 95% CI for the ROR exceeding 1.

### Data Analysis

A descriptive analysis of the clinical characteristics of reports with ICI-related AEs was conducted after screening. The outcome of death reported was included in the fatal group, while others were identified in the nonfatal group. The analysis of time to onset used cases that had a completed START_DT (the date when therapy commenced) recorded in the THER files along with EVENT_DT (the date on which AEs occurred) documented in the DEMO files. To appropriately account for right truncation in estimating the time to onset of AEs from spontaneous reporting databases, we defined an analysis period of 365 days following the commencement of drug administration. The statistical analysis of time to onset was performed with the Weibull Shape Parameter (WSP) test, which assesses the likelihood of changes in AE occurrence over time [22-24]. Specifically, when the shape parameter β is less than 1 and its 95% CI is also less than 1, this indicates a decreasing hazard of AEs over time. Conversely, if β is approximately equal to 1 with a 95% CI that includes 1, this suggests that the hazard remains constant. Furthermore, when β exceeds 1 along with a 95% CI greater than 1, this signifies an escalating hazard over time. Cumulative distribution curves were performed to illustrate the event-to-onset data for ICI-related AEs in different groups.

### Statistical Analysis

Descriptive statistics were applied to summarize the demographic and administrative features of cases involving ICI-related AEs. Categorical variables are presented as frequencies and percentages, compared using chi-square or Fisher exact test. The human anatomy heatmap was conducted with the MOAHIT web tool [25]. Statistical analysis was conducted using R software (version 4.3.0; R Foundation for Statistical Computing) in this study. In this study, statistical significance was determined at the *P*<.05 level.

### Ethical Considerations

This research uses the publicly available and anonymized FAERS database. As this database contains no personally identifiable information and the study does not involve experiments on human participants, it qualifies for exemption from institutional review board assessment and approval.

## Results

### ICI-Related AEs in the Patients With TETs in the FAERS, 2016 Q1 to 2024 Q4

**Figure 1.**
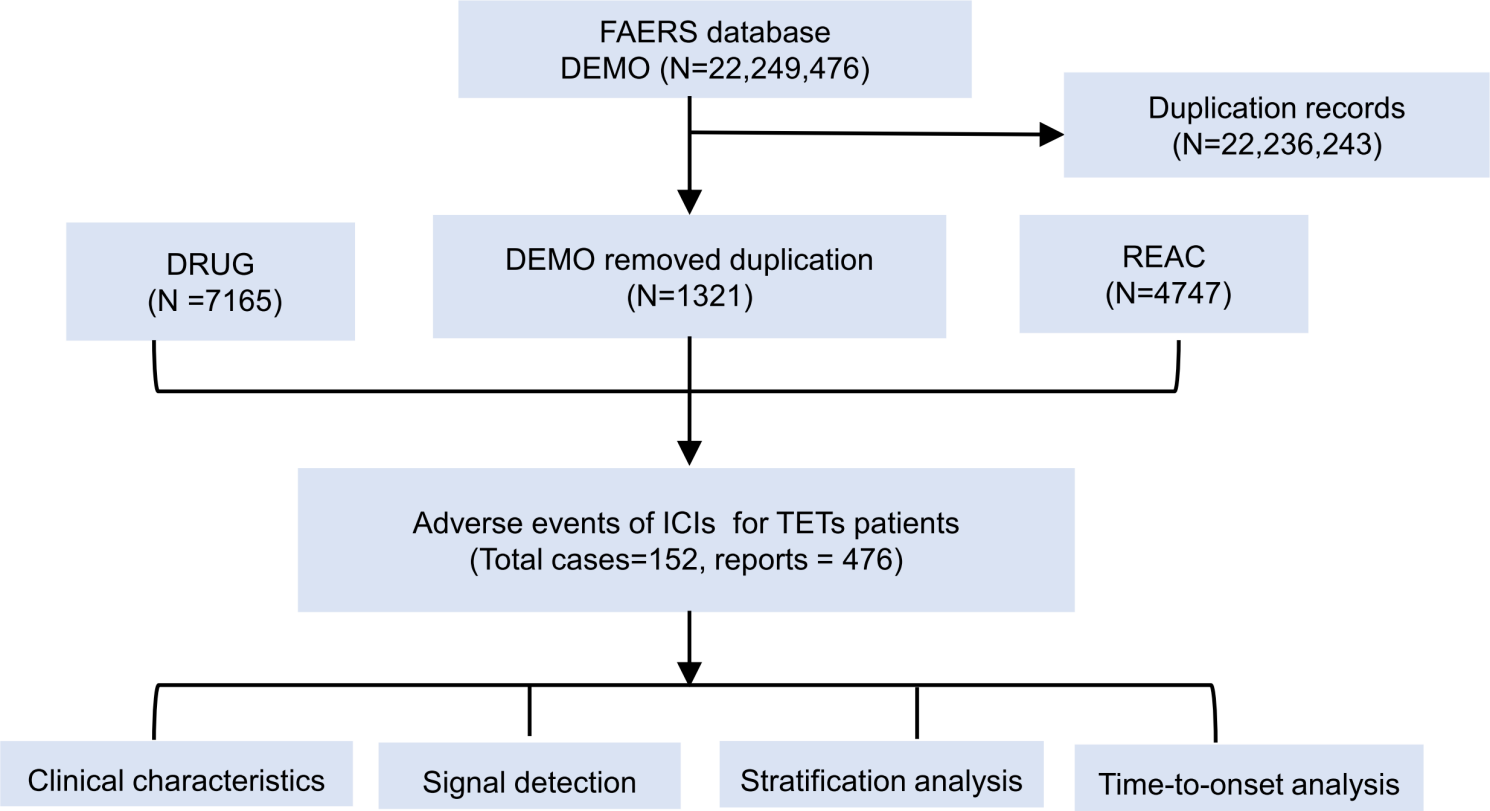
Flowchart showing the analysis process of the study. A detailed description of the selection process of adverse events for ICIs in the FAERS. FAERS: US Food and Drug Administration Adverse Event Reporting System; ICIs: Immune checkpoint inhibitors; TETs: thymic epithelial tumors.

We first investigated the amount of irAEs among patients diagnosed with TETs in the FAERS database. The comprehensive data processing procedure of this study is shown in Figure 1. From the first quarter of 2016 to the fourth quarter of 2024, the FAERS database cataloged a total of 22,249,476 AE reports. The number of ICI-related reports has experienced an increase since 2016 and the proportion of irAEs also varied among different ICI treatment strategies (Figure S1 in Multimedia Appendix 2).

After excluding duplicate cases, 152 cases with TETs who experienced irAEs were identified. The clinical characteristics of patients are summarized in Table 1. Among the total study population with TETs, males accounted for 52.6% (80/152) of cases experiencing irAEs, compared with 40.8% (62/152) in females. Our results indicated that patients aged 18-64 years demonstrated a higher prevalence, accounting for 47.4% (72/152), with the median age of 58.5 years. The majority of cases were sourced from the United States (51/152, 33.6%), followed by Japan (16/152, 10.5%), France (14/152, 9.2%), and China (14/152, 9.2%), with the remaining reports (51/152, 37.5%) coming from 22 other countries. About 77.6% (118/152) of the cases were submitted by health care professionals, while 22.4% (34/152) were reported by consumers (Table 1). We found that 66.4% (101/152) of patients were associated with PD-1 inhibitor treatment strategies, 25.7% (38/152) with PD-L1 inhibitors, and 7.9% (12/152) in the group of the combination therapy of PD-1 inhibitors plus cytotoxic T-lymphocyte–associated antigen 4 inhibitors. Notably, a total of 85.5% (130/152) cases with irAEs were reported in patients receiving ICIs therapy, while 14.5% (22/152) were related to the use of immunotherapy in combination with chemotherapy or targeted agents.

**Table 1.** Characteristics of reports with immune checkpoint inhibitor–related adverse events sourced from the US Food and Drug Administration Adverse Event Reporting System database (January 1, 2011, to December 31, 2024).

Clinical characteristics	Fatal (n=36)	Nonfatal (n=116)	Total (N=152)	*P* value	
Sex, n (%)				.038	
Male	16 (44.4)	64 (55.2)	80 (52.6)		
Female	20 (55.6)	42 (36.2)	62 (40.8)		
Missing	0 (0.0)	10 (8.6)	10 (6.6)		
Age (years), n (%)				.135	
18‐64	18 (50.0)	54 (46.6)	72 (47.4)		
65‐74	3 (8.3)	28 (24.1)	31 (20.4)		
≥75	3 (8.3)	6 (5.2)	9 (5.9)		
Missing	12 (33.3)	28 (24.1)	40 (26.3)		
Age (years), median	53	61	58.5		
Weight, n (%)				.100	
<70	8 (22.2)	32 (27.6)	37 (25.2)		
≥70	3 (8.3)	25 (21.6)	28 (19.0)		
Missing	25 (69.4)	59 (51.8)	82 (55.8)		
Time to onset (days)					
Mean (SD)	57.0 (35.6)	73.2 (26.5)	71.2 (23.6)	.880	
Median (minimum-maximum)	18.0 (8-266)	21.0 (2-1250)	21.0 (2-1250)		
Missing, n (%)	29 (80.1)	66 (56.9)	92 (62.6)		
Disease, n (%)				.489	
Thymomas	15 (41.7)	46 (39.7)	61 (40.1)		
Thymic carcinomas	21 (58.3)	70 (60.3)	91 (59.9)		
Thymic neuroendocrine tumors	0	0	0		
Country, n (%)				.003	
United States	12 (33.3)	39 (33.6)	51 (33.6)		
Japan	0 (0)	16 (13.8)	16 (10.5)		
China	1 (2.8)	13 (11.2)	14 (9.2)		
France	2 (5.6)	12 (10.3)	14 (9.2)		
Spain	1 (2.8)	6 (5.2)	7 (4.6)		
Korea, South	5 (13.9)	2 (1.7)	7 (4.6)		
Other country	15 (41.7)	28 (24.1)	43 (28.3)		
Treatment strategy, n (%)				.012	
Anti-PD-1^a^	29 (80.6)	72 (62.1)	101 (66.4)		
Anti-PD-L1^b^	3 (8.3)	36 (31.0)	39 (25.7)		
Anti-CTLA4^c^	0 (0)	0 (0)	0 (0)		
Combination therapy	4 (11.1)	8 (6.9)	12 (7.9)		
Reported type, n (%)				.250	
Consumer	10 (27.8)	24 (20.7)	34 (22.4)		
Health care professional	26 (72.2)	92 (79.3)	118 (77.6)		

a PD-1: programmed death-1.

b PD-L1: programmed death-ligand 1.

c CTLA4: cytotoxic T-lymphocyte–associated antigen 4.

### Descriptive Analysis of Cases With ICI-Related AEs

**Figure 2.**
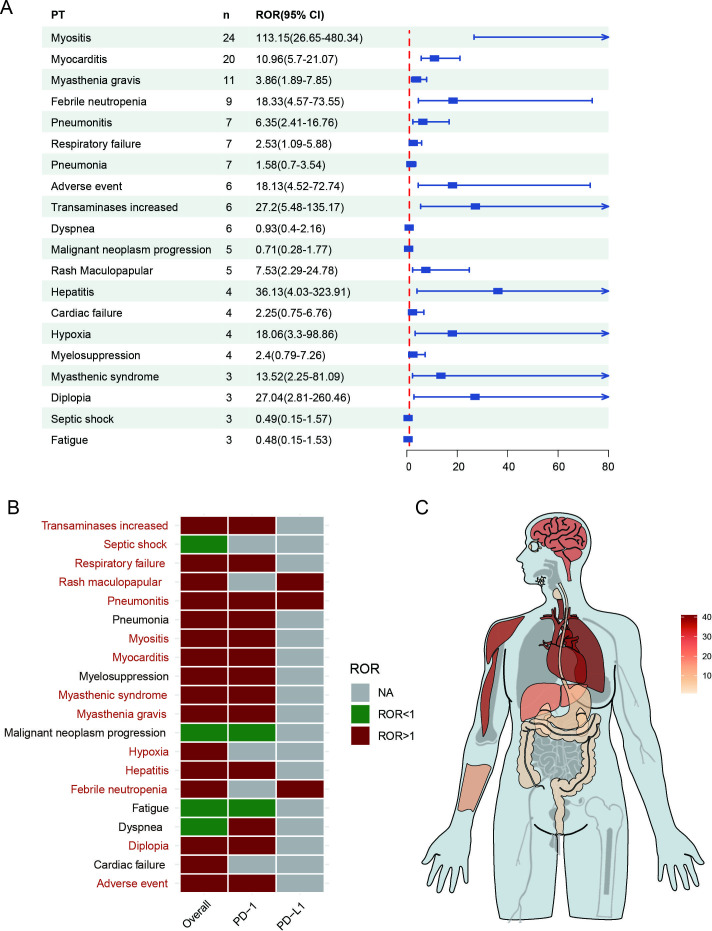
Descriptive analysis of reports with immune-related adverse events (irAEs). (A) A forest plot shows the ROR of the top 20 irAEs. (B) The heatmap shows the ROR for top 20 irAEs (with cases no less than 3) in the US Food and Drug Administration Adverse Event Reporting System database under different immune checkpoint inhibitor treatment strategies (including overall situation, anti-PD-1, and anti-PD-L1). Red indicates ROR values greater than 1, green indicates ROR values less than 1, and gray indicates ROR values that could not be calculated. A significant association signal was labeled in dark red when the lower limit of the 95% CI for the ROR was greater than 1 and the number of cases was at least 3. (C) The anatomical diagram of irAEs occurrence site and the number of cases in patients with thymic epithelial tumors. The darker the color, the more cases with irAEs form this site. NA: not available; PD-1: programmed death-1; PD-L1: programmed death-ligand 1; PT: preferred term; ROR: reporting odds ratio.

We analyzed the categories and number of irAEs reported in patients with TETs treated with ICIs, and a total of 476 AEs were reported in 152 cases of TETs. To further investigate these irAEs, the disproportionality analysis was conducted using the full FAERS database as a reference by calculating ROR of PTs with at least 3 reported cases and the lower limit of the 95% CI for the ROR more than 1. About 14 specific PTs were considered as valid and strongly associated with ICI therapies, covering 11 System Organ Classes. Respiratory, thoracic, and mediastinal disorders were present in 9.03% (43/476) of reports, while cardiac disorders and musculoskeletal and connective tissue disorders were present in 8.19% (39/476) and 7.77% (37/476) of reports, respectively (Table S1 in Multimedia Appendix 2). In addition, myositis (24/476, 5.04%), myocarditis (20/476, 4.20%), myasthenia gravis (11/476, 2.31%), febrile neutropenia (9/476, 1.89%), and pneumonitis were among the top 20 of ICI-related AEs (Table 2). The frequent irAEs of TETs comprised myositis (n=24; ROR 113.15, 95% CI 26.65‐480.34), myocarditis (n=20; ROR 10.96, 95% CI 5.70‐21.07), myasthenia gravis (n=11; ROR 3.86, 95% CI 1.86‐7.85), and febrile neutropenia (n=9; ROR 18.33, 95% CI 4.57‐73.55) (Figure 2A). Among the ICI treatment strategies, anti-PD-1 accounts for the highest proportion at 66.4% (101/152), while anti-PD-L1 and combination therapies account for 25.7% (39/152) and 7.9% (12/152), respectively (Table S2 in Multimedia Appendix 2). The analysis revealed that different irAEs were associated with different ICIs treatment strategies (Figure 2B). The most common AEs of anti-PD-1 agents included myositis (n=18; ROR 56.68, 95% CI 19.08‐168.42), myocarditis (n=17; ROR 6.37, 95% CI 6.37‐25.38), and myasthenia gravis (n=9; ROR 4.23, 95% CI 1.97‐9.09). The most common AEs of anti-PD-L1 agents included febrile neutropenia (n=9; ROR 15.09, 95% CI 6.99‐32.57), rash maculopapular (n=4; ROR 29.12, 95% CI 8.38‐101.24), and pneumonitis (n=3; ROR 10.79, 95% CI 3.05‐38.18). The ICI-related AEs occurrence sites and the number of cases in patients with TETs are shown in the anatomical diagram of Figure 2C.

**Table 2.** The top 20 preferred terms of immune checkpoint inhibitor–related adverse events in patients with thymic epithelial tumors.

PT^a^	n (%)	SOC^b^
Myositis	24 (5.04)	Musculoskeletal and connective tissue disorders
Myocarditis	20 (4.20)	Cardiac disorders
Myasthenia gravis	11 (2.31)	Nervous system disorders
Febrile neutropenia	9 (1.89)	Blood and lymphatic system disorders
Pneumonitis	7 (1.47)	Respiratory, thoracic, and mediastinal disorders
Respiratory failure	7 (1.47)	Respiratory, thoracic, and mediastinal disorders
Pneumonia	7 (1.47)	Infections and infestations
Adverse event	6 (1.26)	General disorders and administration-site conditions
Transaminases increased	6 (1.26)	Investigations
Dyspnea	6 (1.26)	Respiratory, thoracic, and mediastinal disorders
Malignant neoplasm progression	5 (1.05)	Neoplasms benign, malignant, and unspecified (including cysts and polyps)
Rash maculopapular	5 (1.05)	Skin and subcutaneous tissue disorders
Hepatitis	4 (0.84)	Hepatobiliary disorders
Cardiac failure	4 (0.84)	Cardiac disorders
Hypoxia	4 (0.84)	Respiratory, thoracic, and mediastinal disorders
Myelosuppression	4 (0.84)	Blood and lymphatic system disorders
Myasthenic syndrome	3 (0.63)	Nervous system disorders
Diplopia	3 (0.63)	Eye disorders
Septic shock	3 (0.63)	Infections and infestations
Fatigue	3 (0.63)	General disorders and administration-site conditions

a PT: preferred term.

b SOC: System Organ Class.

### Time-to-Onset Analysis

**Figure 3.**
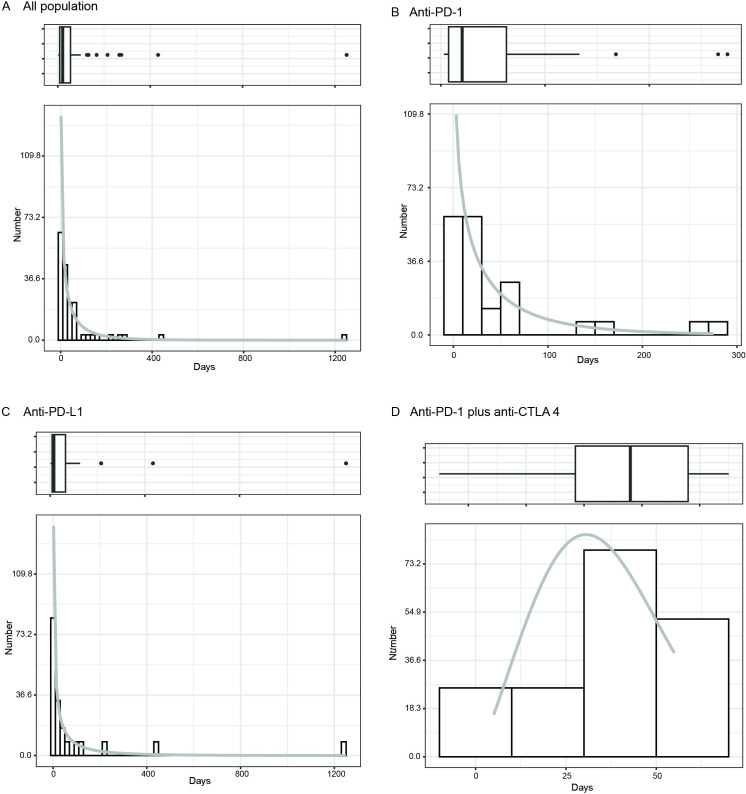
Density plot of the onset time of cases with immune checkpoint inhibitor–related adverse events in different populations with thymic epithelial tumor. (A) Density plot of all populations. (B) Density plot of patients with anti-PD-1 treatments. (**C**) Density plot of patients with anti-PD-L1 treatments. (D) Density plot of patients with anti-PD-1 plus anti-CTLA4 treatments. CTLA4: cytotoxic T-lymphocyte–associated antigen 4; PD-1: programmed death-1; PD-L1: programmed death-ligand 1.

Among the 152 ICI-related cases, 57 patients contained time-to-onset data. The cumulative distribution curves for the onset time of irAEs in different subgroups are present in Figure S2 in Multimedia Appendix 2. The median onset time of irAEs was significantly shorter in patients treated with ICIs plus chemotherapy than in those treated with ICIs alone (9.5 days vs 27.0 days; *P*=.005). No significant difference was present in the subgroup of age, gender, or the specific ICI treatment regimens on the onset time of irAEs in patients with TETs. We found that more than 70% of irAEs occurred within the first 2 months of initiating ICI therapies, with a median onset time of 21.0 days. Results of the WSP test for irAEs are summarized in Table S3 in Multimedia Appendix 2. The distribution of irAEs over the course of ICI treatment is shown in Figure 3. In the WSP test for irAEs, the upper limit of 95% CI of the shape parameter of other drugs was less than 1, indicating an early failure-type profile. Analyses of the time-to-onset profiles of irAEs indicated that early failure–type profiles were found in all populations with ICIs (Figure 3A) or subgroup of anti-PD-1 (Figure 3B) or anti-PD-L1 therapy (Figure 3C). The combination therapy group (anti-PD-1 plus anti-CTLA-4) exhibited a random failure pattern (Figure 3D).

### Comparison Between the Fatal and Nonfatal Groups

In cases of thymoma or thymic carcinoma with irAEs, about 23.7% (36/152) of patients experienced fatal outcomes. Although the presence of irAEs may not directly lead to death, analyzing the differences between the fatal and nonfatal groups remained crucial, as it may provide clues to reduce mortality rates. The gender distribution differed significantly between the fatal and nonfatal groups (*P*=.04). The proportion of female patients was higher in the fatal group (20/36, 55.6%) than in the nonfatal group (42/116, 36.2%). A significant difference was found in the application of ICIs treatment strategies between the fatal and nonfatal groups (*P*=.01). In total, 80.6% (29/36) of cases were treated with PD-1 inhibitors in the fatal group, while 62.1% (72/116) in the nonfatal group.

### Cardiotoxicities Associated With ICIs in Patients With Thymomas and Thymic Carcinomas

A total of 29 patients experienced cardiac toxicities among the 152 cases of TETs with irAEs. More than 1 type of ICI-related cardiotoxicities was found in 7 patients. Among 39 cardiotoxicity-related AEs, myocarditis was the most frequent (20/39, 51.3%), followed by cardiac failure (3/39, 7.9%). ICI treatments showed a significant association with myocarditis (ROR 10.96, 95% CI 5.70-21.07), but no significant association was observed with cardiac failure (ROR 1.82, 95% CI 0.52-6.30). Among the 20 patients diagnosed with TETs who experienced immune-related myocarditis, 11 were female, representing a significant proportion of this subgroup. The median age of these patients was 58 years, and 95.0% (19/20) of patients received PD-1 inhibitor treatment. ICI-related myocarditis was more predominantly observed in patients with thymoma, accounting for 75% (15/20) of cases, and occurs in 25% (5/20) of those with thymic carcinoma (Table S4 in Multimedia Appendix 2). Among the 20 immune-related myocarditis cases, only 6 reports contained time-to-onset data, with the median time of 7 days.

## Discussion

### Principal Findings

This large-scale pharmacovigilance study, using the FAERS database from 2016 to 2024, systematically characterizes the safety profile of ICIs in the patients with TETs. Among 152 identified cases, we observed a distinct and severe spectrum of irAEs. Disproportionality analysis revealed strong signals for myositis (ROR 113.15, 95% CI 26.65‐480.34), myocarditis (ROR 10.96, 95% CI 5.70‐21.07), febrile neutropenia (ROR 18.33, 95% CI 4.57‐73.55), and myasthenia gravis (ROR 3.86, 95% CI 1.86‐7.85). The irAEs profile differed between ICI subclasses, with PD-1 inhibitors predominantly associated with myotoxic and cardiac events, while PD-L1 inhibitors showed stronger signals for febrile neutropenia and rash. Notably, 23.7% (36/152) of reported cases had fatal outcomes, and the median time to irAEs onset was 21 days, with more than 70% of events occurring within the first 2 months of therapy, significantly earlier when ICIs were combined with chemotherapy. These findings underscore a unique and concerning toxicity pattern for ICIs in TETs, dominated by early, severe neuromuscular, cardiac, and hematological complications. The findings will provide crucial insights for the clinical management of ICI therapy in patients with TETs, guiding safer and more effective treatment strategies.

### Comparison With Prior Work

ICIs are now widely used in the treatment of various tumors [6,7]. In long-term follow-up of 40 patients with thymic carcinoma treated with pembrolizumab, an ORR of 22.5%, a median duration of response of 35.9 months, and a 5-year survival rate of 18% were observed [12]. In an open-label phase II trial, pembrolizumab showed encouraging antitumor activity in patients with advanced TETs, with the median PFS of 6 months [26]. Several clinical trials indicated that patients with TETs experienced varying degrees of clinical efficacy from the treatment with PD-1 and PD-L1 inhibitors [11,13,14]. As we all know, tumors escape the surveillance and elimination of the host immune system by activating immune checkpoints, which in turn suppress the cytotoxic killing effect of T cells. By blocking the interaction between immune checkpoints and their ligands, immune checkpoint drugs relieve the inhibition imposed on immune cells, thereby reactivating them to fight against cancer. However, the use of ICIs can also adversely affect normal cells, leading to the disruption of self-tolerance, inducing the development of autoimmune diseases in the body. In a systematic study of toxicities associated with cancer immunotherapy, about 81.15% (6959/8576) of patients had at least 1 toxicity event of varying degrees, and 29.27% (2510/8576) of cases had at least 1 toxicity event at grades 3‐4 [27]. Hematologic toxicities, the most common types of grades 3‐4 toxicities, were present in 9.41% (807/8576) of cases, followed by gastrointestinal toxicity in 5.34% (458/8576) of patients. For toxicities at any grade, patients experiencing respiratory toxicity were at a higher rate of treatment discontinuation than those with gastrointestinal toxicity or hematologic toxicity. However, compared with other cancers, there were certain differences in the terms of irAEs in patients with TETs. Although a meta-analysis reported that ICIs may bring clinical advantages for patients with thymomas, the advantages are limited by the excessively high risk of toxicity [28]. Grades 3‐5 irAEs were reported in 26.4% for patients with TETs, with proportions of 17.1% for thymic carcinoma and 58.3% for thymoma [28]. The most common grade of ≥3 irAEs in TETs included myocarditis, hepatitis, myositis, and myasthenia gravis [12,26,28]. Consistent with reported studies, within our analysis of 476 AEs reports associated with ICIs, myositis and myocarditis were the common AEs, identified in 5.04% (24/476) and 4.20% (20/476) of the cases, respectively. Interestingly, there are differences between thymoma and thymic carcinoma in terms of irAEs in patients with TETs treated with ICIs [29]. Patients with thymoma tend to have a higher incidence of irAEs than those with thymic carcinoma [30]. In patients treated with ICIs, grade 3 or 4 irAEs were reported in 15.4% (4/26) of thymic carcinoma cases compared with 71.4% (5/7) of thymoma cases [26]. They suggested that ICIs should be avoided in patients with thymomas and should be considered with careful monitoring in those with thymic carcinoma. Patients with thymoma who receive ICIs are at considerable risk of experiencing severe immune-mediated toxicity, often presenting with multiple irAEs simultaneously, which is not frequently seen in patients with other cancers receiving ICIs [31].

However, there are several researchers who have been dedicated to unraveling the mechanisms behind the distinct irAEs observed in TETs following the treatment of ICIs. The thymus, a crucial immune organ, is integral to the development and maturation of T cells. It is fundamental in maintaining immune tolerance by generating self-tolerant T cells through positive and negative selection processes [32]. In the context of tumorigenesis, the expression of autoimmune regulator and major histocompatibility complex in thymic epithelial cells declines, resulting in structural alterations within the thymus and impairing immune tolerance mechanisms [33]. This disruption can lead to an increase in immature CD8+ T cells and a decrease in CD4+ T cells and regulatory T cells, consequently promoting the development of autoimmune diseases [34,35]. TETs can promote the release of autoreactive T cells, disturbing peripheral homeostasis and predisposing patients to autoimmunity or causing autoimmune diseases. Some cytokines and chemokines produced by tumor cells induce cross-reactions between tumor antigens and tissue-specific antigens, promoting autoantibody production [29,36]. Furthermore, the administration of ICIs impacts central immune tolerance and disrupts the homeostasis of the peripheral T-cell receptor repertoire, leading to the activation of autoreactive T cells. This disruption can trigger autoimmune diseases in susceptible individuals [37].

ICI-induced myocarditis is a rare immune-related AE in various cancers, occurring in approximately 1% of patients undergoing treatment. However, it is noteworthy for its exceptionally high fatality rate, ranging between 30% and 50% [38-40]. There is limited knowledge on risk factors for ICI myocarditis, with only the combination therapy involving ICIs identified as a reliable risk factor of this condition [41,42]. In addition, the frequency of ICI myotoxicity has been reported 10‐ to 30-fold higher in patients with TETs than in those with other cancers [43]. The seriousness of several life-threatening ICI-related myotoxicity was demonstrated in young patients with thymoma, who experienced a fatal triad of fulminant myocarditis, myositis, and a myasthenia gravis–like syndrome, emerging within a few days following a single dose of ICI treatment [44]. In line with our findings, the median time to onset of myocarditis was 7 days after the administration of immunotherapy. ICI-related myocarditis in patients with TETs tends to occur earlier and more severely after the initiation of ICI treatment than other cancers, resulting in life-threatening arrhythmias. Our findings revealed that the ICI-related myocarditis in patients with TETs was observed more in females and those with the treatment of anti-PD-1 inhibitors or the presence of thymomas. Emerging evidence indicated that autoreactive T cells recognizing the cardiac antigen α-actinin played a crucial role in the development of ICI-related myocarditis, unveiling the key mechanisms behind ICI-related myocarditis [45]. Increased frequencies and spatial colocalization of cytotoxic T lymphocytes, conventional dendritic cells, and inflammatory fibroblasts have been shown in ICI-related myocarditis heart tissue. In addition, the presence of heart-expanded T-cell receptors in a cycling blood CD8+ T-cell population may indicate whether a patient’s myocarditis could potentially lead to death [46]. For patients with TETs who experienced myocarditis, research indicated that thymus-related dysfunction was linked to the incidence and severity of ICI-induced myotoxicities, including myocarditis and myositis, and identifying TETs and assessing acetylcholine receptor through radiobiological workup may help predict and stratify the risk of severe ICI myotoxicities in patients with cancer undergoing ICI treatment [43].

### Strengths and Limitations

We conducted a relatively large-scale, disease-specific pharmacovigilance analysis of irAEs in patients with TETs in the real-world setting. This study addressed a significant knowledge gap in the field, as TETs originate from the central immune organ, thereby establishing a distinctive and high-risk immunological context for ICIs toxicity that is often obscured in broader, pan-cancer analyses. The findings provide essential evidence to inform clinical guidelines for ICIs use in TETs, representing a significant contribution to a niche yet high-stakes area of oncology. By systematically delineating the unique toxicity profile, our work has the potential to impact both clinical decision-making and design of future studies in TETs.

There were several limitations in this research. First, the data were sourced from a spontaneously reported database, which is subject to known biases such as underreporting, duplicate reports, and confounding factors, potentially affecting data quality and completeness [47]. Moreover, reports in the FAERS database predominantly originate from North America and Europe, resulting in a relatively low representation of Asian populations. Second, the use of ROR, although valuable for signal detection, may inflate associations for established class-specific AEs, such as myocarditis, myositis, and pneumonitis in the context of ICIs, due to heightened clinical awareness and targeted reporting. Consequently, ROR-based signals for known irAEs should be interpreted cautiously, as they largely reflect anticipated class effects rather than novel risks. Furthermore, the signal strength reflects relative risk and does not allow for the quantification of absolute risk. Third, the comparative analysis between anti-PD-1 and anti-PD-L1 agents should be interpreted with caution. While the differential toxicity signals provide hypothesis-generating insights, the clinical application of PD-L1 inhibitors in TETs remains limited. Consequently, the smaller sample size for PD-L1 cases in this study may reduce the statistical robustness of direct comparisons and limit the generalizability of subclass-specific findings. Finally, it is of critical clinical importance to understand the factors that determine who may develop irAEs. Due to the limited number of cases with time-to-onset data, the study could not conduct univariate or multivariate regression analyses for irAEs, which represents an important gap for clinical risk stratification. In addition, these findings are hypothesis-generating and require validation through well-designed epidemiological studies to clarify incidence rates, underlying mechanisms, and real-world clinical relevance of the identified signals.

### Clinical Implications and Future Directions

The findings from this pharmacovigilance analysis carry significant clinical implications for the management of patients with TETs receiving ICI therapy. The robust and early-onset signals for severe toxicities, particularly myositis and myocarditis, necessitate a paradigm shift in monitoring protocols. We recommend implementing proactive, protocol-driven monitoring within the first 2 months of treatment initiation for this high-risk population. Furthermore, the high fatality rate associated with cardiotoxicity, especially in specific subgroups, underscores the critical need for multidisciplinary management involving cardio-oncology specialists.

Future research should be directed toward translating these hypothesis-generating signals into actionable clinical knowledge. First, prospective cohort studies are needed to establish accurate incidence rates and validate risk stratification models incorporating clinical and molecular biomarkers. Second, mechanistic investigations should explore the unique immunobiology of TETs that predisposes patients to these severe toxicities. Third, there is an urgent need to develop TETs-specific clinical guidelines for irAE prevention, monitoring, and management, which should include standardized baseline assessment protocols and treatment algorithms tailored to this population’s distinct risk profile.

### Conclusions

This large-scale pharmacovigilance analysis, using real-world data from the FAERS database, delineates irAEs profile associated with ICIs therapy in patients with TETs. The study identified strong disproportionate reporting signals for myositis, myocarditis, myasthenia gravis, and febrile neutropenia, with more than 70% of irAEs occurring within the first 2 months of treatment initiation, with a median onset time of 21.0 days. As a hypothesis-generating investigation, these findings emphasize the need for vigilant early monitoring and support tailored surveillance strategies in clinical practice, particularly in patients with thymoma. Further research is warranted to elucidate underlying mechanisms, identify predictive biomarkers, and develop risk mitigation approaches to optimize the safety and efficacy of immunotherapy in this population.

## Supplementary material

10.2196/76908 Multimedia Appendix 1 Details on the US Food and Drug Administration Adverse Event Reporting System database and the Medical Dictionary for Regulatory Activities terminology used in the analysis.

10.2196/76908 Multimedia Appendix 2 Supplementary figures and tables supporting the analysis of immune checkpoint inhibitor–related adverse events in patients with thymic epithelial tumors.

## Abbreviations

AE: adverse event
FAERS: US Food and Drug Administration Adverse Event Reporting System
FDA: Food and Drug Administration
ICI: immune checkpoint inhibitor
irAEs: immune-related adverse events
MedDRA: Medical Dictionary for Regulatory Activities
ORR: objective response rate
PD-1: programmed death-1
PD-L1: programmed death-ligand 1
PFS: progression-free survival
PT: preferred term
ROR: reporting odds ratio
TET: thymic epithelial tumor
WSP: Weibull Shape Parameter

## References

[1] Marx A, Chan JKC, Chalabreysse L (2022). The 2021 WHO classification of tumors of the thymus and mediastinum: what is new in thymic epithelial, germ cell, and mesenchymal tumors?. J Thorac Oncol.

[2] Remon J, Bernabé R, Diz P (2022). SEOM-GECP-GETTHI Clinical Guidelines for the treatment of patients with thymic epithelial tumours (2021). Clin Transl Oncol.

[3] Multidisciplinary Committee of Oncology, Chinese Physicians Association (2021). Chinese guideline for clinical diagnosis and treatment of thymic epithelial tumors (2021 Edition). Zhonghua Zhong Liu Za Zhi.

[4] Conforti F, Pala L, Giaccone G, De Pas T (2020). Thymic epithelial tumors: from biology to treatment. Cancer Treat Rev.

[5] Girard N, Ruffini E, Marx A, Faivre-Finn C, Peters S (2015). Thymic epithelial tumours: ESMO Clinical Practice Guidelines for diagnosis, treatment and follow-up. Ann Oncol.

[6] Pardoll DM (2012). The blockade of immune checkpoints in cancer immunotherapy. Nat Rev Cancer.

[7] Sanmamed MF, Chen L (2018). A paradigm shift in cancer immunotherapy: from enhancement to normalization. Cell.

[8] Gibney GT, Weiner LM, Atkins MB (2016). Predictive biomarkers for checkpoint inhibitor-based immunotherapy. Lancet Oncol.

[9] Zhang Y, Zhang Z (2020). The history and advances in cancer immunotherapy: understanding the characteristics of tumor-infiltrating immune cells and their therapeutic implications. Cell Mol Immunol.

[10] Xu C, Zhang Y, Wang W (2023). Chinese expert consensus on the diagnosis and treatment of thymic epithelial tumors. Thorac Cancer.

[11] Giaccone G, Kim C (2021). Durable response in patients with thymic carcinoma treated with pembrolizumab after prolonged follow-up. J Thorac Oncol.

[12] Giaccone G, Kim C, Thompson J (2018). Pembrolizumab in patients with thymic carcinoma: a single-arm, single-centre, phase 2 study. Lancet Oncol.

[13] Katsuya Y, Horinouchi H, Seto T (2019). Single-arm, multicentre, phase II trial of nivolumab for unresectable or recurrent thymic carcinoma: PRIMER study. Eur J Cancer.

[14] Rajan A, Heery CR, Thomas A (2019). Efficacy and tolerability of anti-programmed death-ligand 1 (PD-L1) antibody (Avelumab) treatment in advanced thymoma. J Immunother Cancer.

[15] Thapa P, Farber DL (2019). The role of the thymus in the immune response. Thorac Surg Clin.

[16] Jayathilaka B, Mian F, Franchini F, Au-Yeung G, IJzerman M (2025). Cancer and treatment specific incidence rates of immune-related adverse events induced by immune checkpoint inhibitors: a systematic review. Br J Cancer.

[17] Girard N, Ponce Aix S, Cedres S (2023). Efficacy and safety of nivolumab for patients with pre-treated type B3 thymoma and thymic carcinoma: results from the EORTC-ETOP NIVOTHYM phase II trial. ESMO Open.

[18] Hao Y, Lin G, Xiang J (2023). Analysis of the efficacy and safety of immunotherapy in advanced thymoma patients. Cancer Med.

[19] FDA Adverse Event Reporting System (FAERS). US Food & Drug Administration.

[20] Fusaroli M, Salvo F, Begaud B (2024). The Reporting of a Disproportionality Analysis for Drug Safety Signal Detection Using Individual Case Safety Reports in PharmacoVigilance (READUS-PV): development and statement. Drug Saf.

[21] Fusaroli M, Salvo F, Begaud B (2024). The REporting of A Disproportionality Analysis for DrUg Safety Signal Detection Using Individual Case Safety Reports in PharmacoVigilance (READUS-PV): explanation and elaboration. Drug Saf.

[22] Abe J, Umetsu R, Mataki K (2016). Analysis of Stevens-Johnson syndrome and toxic epidermal necrolysis using the Japanese Adverse Drug Event Report database. J Pharm Health Care Sci.

[23] Nakamura M, Umetsu R, Abe J (2015). Analysis of the time-to-onset of osteonecrosis of jaw with bisphosphonate treatment using the data from a spontaneous reporting system of adverse drug events. J Pharm Health Care Sci.

[24] Sauzet O, Carvajal A, Escudero A, Molokhia M, Cornelius VR (2013). Illustration of the Weibull shape parameter signal detection tool using electronic healthcare record data. Drug Saf.

[25] Zhou C, Lin A, Li M, Qi C, Zhang J, Luo P (2023). MOAHIT: a web tool for visualizing tumor multi-omics data with human anatomy heatmaps. bioRxiv.

[26] Cho J, Kim HS, Ku BM (2019). Pembrolizumab for patients with refractory or relapsed thymic epithelial tumor: an open-label phase II trial. J Clin Oncol.

[27] Kong X, Chen L, Su Z (2023). Toxicities associated with immune checkpoint inhibitors: a systematic study. Int J Surg.

[28] Remon J, Villacampa G, Facchinetti F (2023). Immune checkpoint blockers in patients with unresectable or metastatic thymic epithelial tumours: a meta-analysis. Eur J Cancer.

[29] Ao YQ, Gao J, Wang S (2023). Immunotherapy of thymic epithelial tumors: molecular understandings and clinical perspectives. Mol Cancer.

[30] Song X, Fan J, Zhu L, Wang Z, He Y, Zhou C (2021). The efficacy and safety of immunotherapy in thymic epithelial tumors: more effective, more risky: a systematic review. J Thorac Dis.

[31] Perrino M, Cordua N, De Vincenzo F (2023). Thymic epithelial tumor and immune system: the role of immunotherapy. Cancers (Basel).

[32] Ashby KM, Hogquist KA (2024). A guide to thymic selection of T cells. Nat Rev Immunol.

[33] Weksler B, Lu B (2014). Alterations of the immune system in thymic malignancies. J Thorac Oncol.

[34] Perrino M, Voulaz E, Balin S (2024). Autoimmunity in thymic epithelial tumors: a not yet clarified pathologic paradigm associated with several unmet clinical needs. Front Immunol.

[35] Xin Z, Lin M, Hao Z (2022). The immune landscape of human thymic epithelial tumors. Nat Commun.

[36] Maniar R, Loehrer PJ (2023). Understanding the landscape of immunotherapy in thymic epithelial tumors. Cancer.

[37] Sutanto H, Safira A, Fetarayani D (2024). From tumor to tolerance: a comprehensive review of immune checkpoint inhibitors and immune-related adverse events. Asia Pac Allergy.

[38] Nguyen LS, Cooper LT, Kerneis M (2022). Systematic analysis of drug-associated myocarditis reported in the World Health Organization pharmacovigilance database. Nat Commun.

[39] Power JR, Alexandre J, Choudhary A (2022). Association of early electrical changes with cardiovascular outcomes in immune checkpoint inhibitor myocarditis. Arch Cardiovasc Dis.

[40] Wang DY, Salem JE, Cohen JV (2018). Fatal toxic effects associated with immune checkpoint inhibitors: a systematic review and meta-analysis. JAMA Oncol.

[41] Johnson DB, Balko JM, Compton ML (2016). Fulminant myocarditis with combination immune checkpoint blockade. N Engl J Med.

[42] Salem JE, Manouchehri A, Moey M (2018). Cardiovascular toxicities associated with immune checkpoint inhibitors: an observational, retrospective, pharmacovigilance study. Lancet Oncol.

[43] Fenioux C, Abbar B, Boussouar S (2023). Thymus alterations and susceptibility to immune checkpoint inhibitor myocarditis. Nat Med.

[44] Nguyen LS, Bretagne M, Arrondeau J (2022). Reversal of immune-checkpoint inhibitor fulminant myocarditis using personalized-dose-adjusted abatacept and ruxolitinib: proof of concept. J Immunother Cancer.

[45] Axelrod ML, Meijers WC, Screever EM (2022). T cells specific for α-myosin drive immunotherapy-related myocarditis. Nature New Biol.

[46] Blum SM, Zlotoff DA, Smith NP (2024). Immune responses in checkpoint myocarditis across heart, blood and tumour. Nature New Biol.

[47] Sakaeda T, Tamon A, Kadoyama K, Okuno Y (2013). Data mining of the public version of the FDA Adverse Event Reporting System. Int J Med Sci.

