# Supplementary material for: Immunotherapy Safety in Thymic Epithelial Tumors: Disproportionality Analysis of the Food and Drug Administration Adverse Event Reporting System
Source: JMIR Cancer. 2026 Feb 25;12:e76908. doi: 10.2196/76908 (PMC12935294; doi:10.2196/76908)
Supplement: Multimedia Appendix 1 [file cancer-v12-e76908-s001.pdf]

Multimedia Appendix 1

Method

The FAERS compiles safety reports on a quarterly basis into a consolidated file. Each consolidated file organizes detailed information about all case reports from that quarter into seven separate tables, which include information on patient demographics and administrative details (DEMO), medications and biological products administered (DRUG), adverse drug reactions (REAC), patient outcomes (OUTC), report sources (RPSR), drug therapy start and end dates (THER), and indications for medication use (INDI). The FAERS classifies the role of each drug in its associated reports as Primary Suspect (PS), Secondary Suspect (SS), Concomitant (C), or Interacting (I).The international Medical Dictionary for Regulatory Activities (MedDRA) is systematically structured into five levels: lowest level term (LTT), PT, high level term (HLT), high level group term (HLGT), and system organ class (SOC). PTs serve as unique descriptors for individual medical concepts, including signs, symptoms, and disease diagnoses. Each PT is assigned to a primary SOC and may also be classified into one or more secondary SOCs.
