# Supplementary material for: Immunotherapy Safety in Thymic Epithelial Tumors: Disproportionality Analysis of the Food and Drug Administration Adverse Event Reporting System
Source: JMIR Cancer. 2026 Feb 25;12:e76908. doi: 10.2196/76908 (PMC12935294; doi:10.2196/76908)
Supplement: Multimedia Appendix 2 [file cancer-v12-e76908-s002.pdf]

Multimedia Appendix 2

Figure S1 Statistics on the occurrence of adverse events in ICIs reports from the FAERS database during 2016–2024. (A) The upper bar plot shows the number of ICIs reports with thymic epithelial tumors in the FAERS database during 2016 and 2024, as well as the overall situation. (B) The proportional bar plot below shows the amount of ICI reports with thymic epithelial tumors for different ICI treatment strategies in the FAERS database from 2016 to 2024 and overall situation. The ICI treatment strategies included anti-CTLA-4 and anti-PD-1, anti-PD-1, and anti-PD-L1.

Figure S1

A

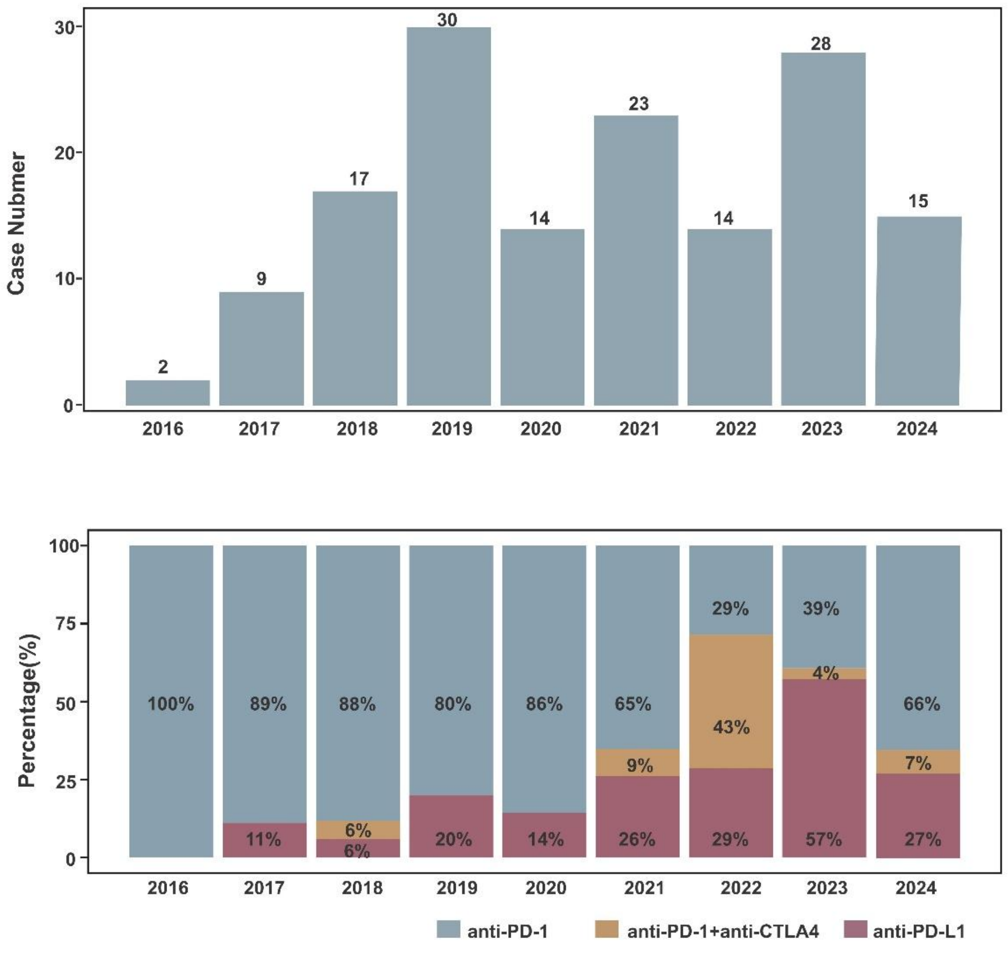

Figure S2 The cumulative distribution curves demonstrate the onset time of ICI-related AE after treatment with ICIs in different subgroups. (A) age; (B) sex; (C) plus chemotherapy or not; (D) fatal status; (E) different treatment strategies. Statistical tests were conducted using the nonparametric Wilcoxon rank sum test. chemo, chemotherapy.

Figure S2

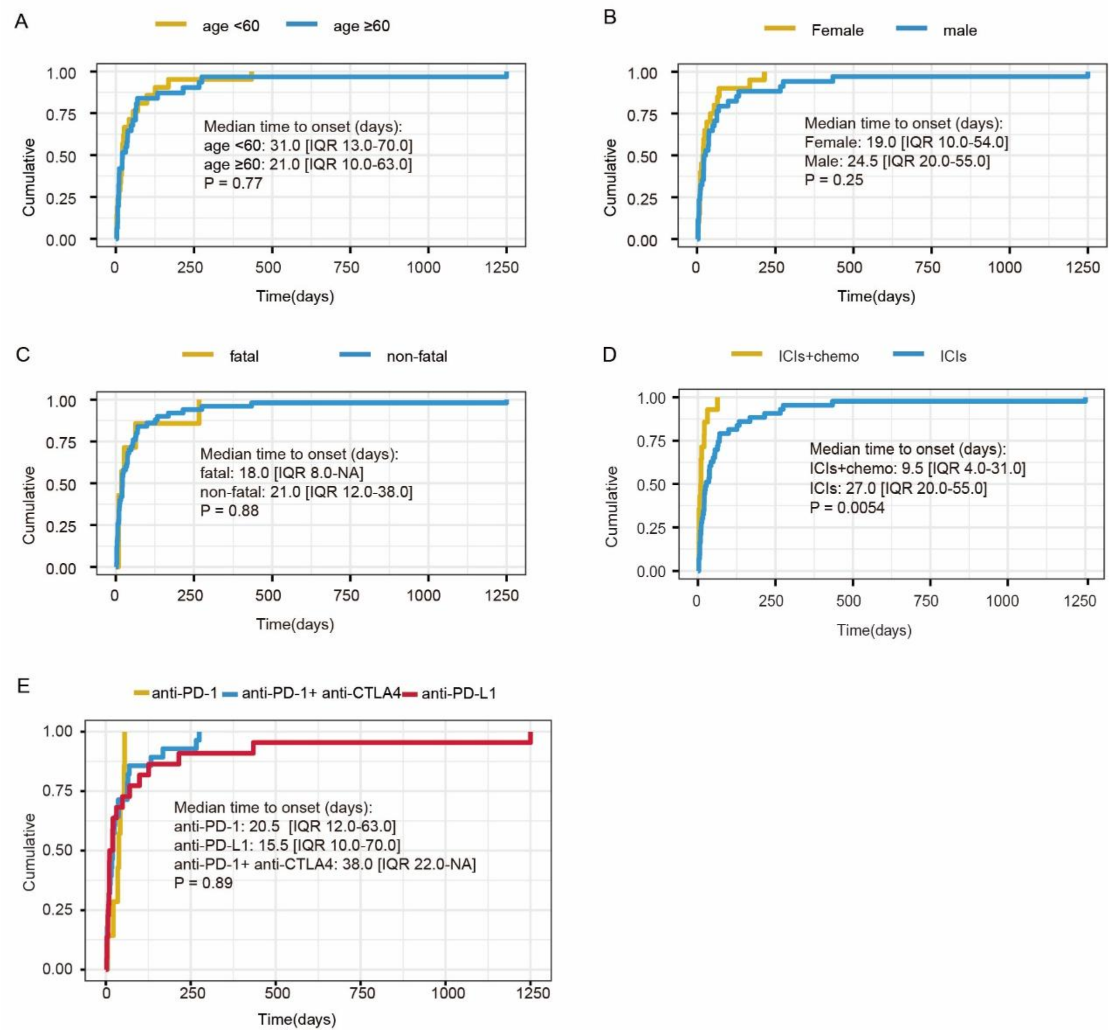

Table S1 The frequency distribution of adverse events by System Organ Class (SOC).

SOC[a]	N (%)
General Disorders and Administration Site Conditions	45 (9.45)
Respiratory, Thoracic and Mediastinal Disorders	43 (9.03)
Cardiac Disorders	39 (8.19)
Musculoskeletal and Connective Tissue Disorders	37 (7.77)
Infections and Infestations	34 (7.14)
Nervous System Disorders	31 (6.51)
Investigations	24 (5.04)
Hepatobiliary Disorders	22 (4.62)
Blood and Lymphatic System Disorders	18 (3.78)
Gastrointestinal Disorders	17 (3.57)
Skin and Subcutaneous Tissue Disorders	16 (3.36)
Neoplasms Benign, Malignant and Unspecified (Incl Cysts and Polyps)	11 (2.31)
Endocrine Disorders	10 (2.10)
Immune System Disorders	8 (1.68)
Eye Disorders	7 (1.47)
Metabolism and Nutrition Disorders	7 (1.47)
Surgical and Medical Procedures	6 (1.26)
Vascular Disorders	6 (1.26)
Renal and Urinary Disorders	4 (0.84)
Psychiatric Disorders	3 (0.63)

[a]SOC: system organ class

Table S2 The number of reports with ICI-related AEs for different ICI treatment strategies.

Drugs	N	Treatment_Strategy
Pembrolizumab	65	Anti-PD-1[a]
Nivolumab	31	Anti-PD-1
Tislelizumab	4	Anti-PD-1
Toripalimab	1	Anti-PD-1
Atezolizumab	24	Anti-PD-L1[b]
Avelumab	10	Anti-PD-L1
Duralumab	5	Anti-PD-L1
Nivolumab+Ipilimumab	12	Anti-PD-1+Anti-CTLA-4[c]

[a]PD-1: programmed death-1

[b]PD-L1: programmed death-ligand 1

[c]CTLA4: cytotoxic T-lymphocyte-associated antigen 4

Table S3 Weibull shape parameter test for the onset profile of ICI-related AEs

	Case reports	Scale parameter (95%CI[a])	Shape parameter (95%CI)	Type
All population	57	54.23 (10.63-97.83)	0.55(0.39-0.72)	Early failure
Anti-PD-1[a]	28	44.40 (20.13-62.68)	0.77(0.56-0.97)	Early failure
Anti-PD-L1[b]	22	54.23(10.63-97.83)	0.55(0.39- 0.72)	Early failure
Anti-PD-1 plus anti-CTLA4[c]	7	40.00(26.17-53.83)	2.22 (0.77-3.67)	Random failure

[a]CI: confidence interval

[b]PD-1: programmed death-1

[c]PD-L1: programmed death-ligand 1

[d]CTLA4: cytotoxic T-lymphocyte-associated antigen 4

Table S4 Characteristics of reports with myocarditis of immunotherapy in the patients with thymic epithelial tumors soured from the FAERS database (Jan 1, 2011 – Dec 31, 2024)

**Clinical characteristics**	**Total (n=20)**
**Sex, n (%)**	
Male	7 (35.0)
Female	11 (55.0)
Missing	2 (10.0)
**Age, n (%)**	
18-64	12 (60.0)
65-74	6 (30.0)
≥75	0 (0)
Missing	2 (10.0)
**Age, Median (years)**	58
**Time to onset (days)**	
Median (Minimum-Maximum)	7 (6-21)
Missing, n (%)	14 (70.0)
**Treatment strategy, n (%)**	
Anti-PD-1	19 (95.0)
Anti-PD-L1	1 (5.0)
Anti-CTLA4	0 (0)
Combination therapy	0(0)
**Disease type, n (%)**	
Thymoma	15 (75.0)
Thymic carcinoma	5 (25.0)
Thymic neuroendocrine tumors	0 (0)
